# Multidirectional Filamented Light Biofabrication Creates Aligned and Contractile Cardiac Tissues

**DOI:** 10.1002/advs.202404509

**Published:** 2024-10-07

**Authors:** Lewis S. Jones, Miriam Filippi, Mike Yan Michelis, Aiste Balciunaite, Oncay Yasa, Gal Aviel, Maria Narciso, Susanne Freedrich, Melanie Generali, Eldad Tzahor, Robert K. Katzschmann

**Affiliations:** ^1^ Soft Robotics Laboratory ETH Zurich Tannenstrasse 3 Zurich 8092 Switzerland; ^2^ Department of Molecular Cell Biology Weizmann Institute of Science Rehovot 76100 Israel; ^3^ Swiss Federal Laboratories for Materials Science and Technology (EMPA) Dubendorf 8600 Switzerland; ^4^ Experimental Continuum Mechanics ETH Zurich Leonhardstrasse 21 Zurich 8092 Switzerland; ^5^ ETH Phenomics Center ETH Zurich Otto‐Stern‐Weg 7 Zurich 8093 Switzerland; ^6^ Institute for Regenerative Medicine (IREM) University of Zurich Schlieren 8952 Switzerland

**Keywords:** cardiac tissue engineering, cardiomyocytes, cell alignment, contractile tissues, volumetric bioprinting

## Abstract

Biofabricating 3D cardiac tissues that mimic the native myocardial tissue is a pivotal challenge in tissue engineering. In this study, we fabricate 3D cardiac tissues with controlled, multidirectional cellular alignment and directed or twisting contractility. We show that multidirectional filamented light can be used to biofabricate high‐density (up to 60 × 10^6^ cells mL^−1^) tissues, with directed uniaxial contractility (3.8x) and improved cell‐to‐cell connectivity (1.6x gap junction expression). Furthermore, by using multidirectional light projection, we can partially overcome cell‐induced light attenuation, and fabricate larger tissues with multidirectional cellular alignment. For example, we fabricate a tri‐layered myocardium‐like tissue and a bi‐layered tissue with torsional contractility. The approach provides a new strategy to rapidly fabricate aligned cardiac tissues relevant to regenerative medicine and biohybrid robotics.

## Graphical abstract

We fabricate 3D cardiac tissues using multidirectional filamented light biofabrication. This method generates microstructures in hydrogels to align cells within highly concentrated bioinks. The resulting tissues exhibit enhanced connectivity and contractility. Furthermore, we use multidirectional light projection to fabricate larger tissues with multidirectional cell alignment and twisting contractility. This approach has implications for regenerative medicine and biohybrid robotics.

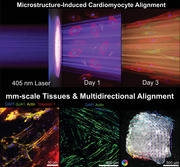

## 1 Introduction

The long‐term goal of cardiac tissue engineering is to recreate the complex structure and function of the native myocardium. The myocardium has three aligned layers of cardiac muscle, transitioning from a helical endocardial (α = −60°) to epicardial layer (α = −60°).^[^ ^1^ ^]^ This muscle shortens to cause an efficient torsional contraction. Therefore, one focus of cardiac tissue engineering is to control cardiomyocyte alignment. Furthermore, researchers have shown that cardiomyocyte alignment can contribute to cardiomyocyte maturity.^[^ ^2^, ^3^ ^]^ In the short term, controlling 3D cardiomyocyte alignment will enable us to fabricate improved tissues for cardiac repair or as substrates for drug screening.

Various manufacturing techniques, such as hydrogel molding^[^ ^4^, ^5^ ^]^ and extrusion‐based bioprinting,^[^ ^6^, ^7^, ^8^, ^9^ ^]^ have been used to fabricate 3D cardiac tissues at high‐cell‐density. Hydrogel molding can be used to fabricate tissues at a near‐unlimited cell seeding density but without microstructures for cellular alignment. However, to align cells using casting, additional techniques are required: for example, structural techniques,^[^ ^10^, ^11^, ^12^ ^]^ mechanical loading,^[^ ^13^, ^14^, ^15^ ^]^ electrical stimulation,^[^ ^16^, ^17^ ^]^ magnetic,^[^ ^18^, ^19^ ^]^ and acoustic fields.^[^ ^20^, ^21^, ^22^ ^]^ Extrusion‐based bioprinting techniques can fabricate cardiac tissues at a physiological cell density^[^ ^23^ ^]^ and induce cellular alignment in 3D tissues (e.g., via shear force).^[^ ^8^, ^9^ ^]^ However, preparing alignment‐inducing additives and layer‐by‐layer fabrication is time‐consuming. Furthermore, the cellular alignment is typically limited to the horizontal axis. Fiber spinning can fabricate increasingly complex cardiac tissue architectures.^[^ ^24^ ^]^ However, this technique is limited to producing symmetric, acellular, and fibrous scaffolds. Furthermore, these scaffolds must be seeded with cells after fabrication, limiting the cell density of the tissue. In summary, current biofabrication techniques cannot freely distribute 3D microscale mechanical cues for cellular alignment in situ, particularly at the cell density required for cardiac tissue contractility (10^6^–10^7^ cells mL^−1^)^[^ ^25^ ^]^ or found in native tissues (10^7^–10^8^ cells mL^−1^).^[^ ^26^, ^27^ ^]^

Previous research has demonstrated that 3D microstructure networks can be formed during the polymerization of photopolymers.^[^ ^28^ ^]^ Modulation instability describes how minor variations (noise) in light amplitude and phase become amplified, causing an initially continuous wave beam to collapse into discrete filaments of light. These filaments are captured in the photopolymer, owing to the self‐focussing effect that occurs during polymerization.^[^ ^29^ ^]^ Recently, Filamented light (FLight) biofabrication was developed to create microfilament structures (2–30 µm) in hydrogels. The resulting microfilaments and interfilament microchannels were shown to align fibroblasts, tenocytes, endothelial cells, and skeletal muscle myoblasts within the bioprinted hydrogels.^[^ ^30^ ^]^ Nevertheless, even a low density of cells in the photoink (4–5 million cells mL^−1^) induces light scattering during bioprinting.^[^ ^31^, ^32^, ^33^ ^]^ Therefore, FLight biofabrication has not yet been demonstrated at near‐physiological bioink cell densities with non‐proliferative cell types, such as cardiomyocytes. Here, high cellular densities are required for functionality, specifically in the case of contractile muscle tissues.

Our work introduces multidirectional FLight biofabrication, which can generate cell‐aligning microfilaments into high‐cell density, optically tuned hydrogels. We specifically biofabricate contractile cardiac tissues with controlled cellular alignment, using bioinks of 15–60 million cells mL^−1^. Furthermore, we explore how high cell‐density biofabrication adversely affects microfilament‐induced cell alignment, causing anisotropic light irradiance, hydrogel stiffness, and remodeling. We use multidirectional FLight biofabrication to overcome these limitations, which stabilizes the engineered tissues and can create complex multidirectional cell alignments. Specifically, we biofabricate tissues based on the structure of a jellyfish and tri‐layered myocardium. Finally, we benchmark our microstructured tissues against unstructured cardiac tissues, showing higher levels of protein alpha 1 (GJA1) (1.6x) and distinctive uniaxial contractility (3.8x, 27 µm vs 7.2 µm). Our work is the first example of engineering bioinspired, contractile cardiac tissues using FLight biofabrication and lays the foundation to use this technology for regenerative medicine or biohybrid robotics.

## 2 Results

### 2.1 Filamented Light Biofabrication of Cardiac Tissues in Optically Tuned Hydrogels

**Figure 1:**
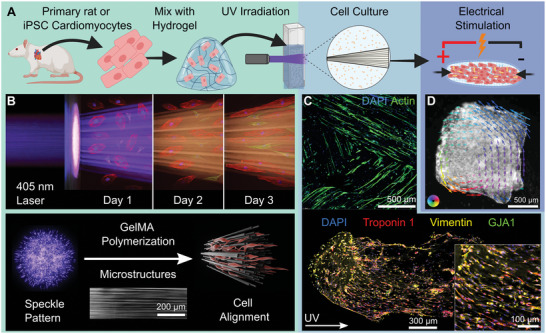
Aligned and contractile cardiac tissues are fabricated at a physiological cell density using filamented light biofabrication. A) Schematic illustration showing cardiac tissue biofabrication, cell culture, and electrical stimulation. B) Schematic illustration of filamented light biofabrication showing cell‐induced light scattering, cell alignment, microstructure formation, and cardiomyocyte (red) and fibroblast (yellow) alignment. Hydrogel microstructures form when the laser speckle pattern is nonlinearly amplified (self‐focussing) due to photoink crosslinking, resulting in microscopic waveguides. C) Immunofluorescence staining of cells in a biomimetic tri‐layered myocardium tissue (multidirectional light) and unidirectionally irradiated tissue (directional light). D) Optical flow contractility analysis on a tissue created with multidirectional filamented light biofabrication.

We used FLight to biofabricate microstructured GelMA (Gelatin Methacrylate) hydrogels containing primary cardiac cells, or iPSC‐CMs (induced Pluripotent Stem Cell Cardiomyocytes) (**Figure** **1**). Our primary cell mixture is enriched for cardiomyocytes using Magnetic Cell Sorting (MACS) but also contains non‐myocyte cell types (fibroblasts and endothelial cells).^[^ ^34^ ^]^ We projected an image containing a circle (ø 500 µm) on bioinks at high cell density (15, 30, 45, and 60 × 10^6^ cells mL^−1^). The circular projection formed contractile cylindrical cardiac tissues (ø 0.6 mm x 2 mm) (Figure 1A). The speckle pattern from the coherent UV light undergoes self‐focusing due to photoink crosslinking, forming polymerized microfilaments and unpolymerized microchannels, which induce cell alignment (Figure 1B; Videos S1 and S2, Supporting Information). Following 4–5 days in cell culture, we observed the alignment of cardiomyocytes and fibroblasts (Figure 1C) and distinctive directional tissue contractility (Figure 1D).

During preliminary experiments, we aimed to maximize the cell density of the bioink without adversely affecting the formation of hydrogel microstructures. However, cell‐induced light scattering reduced the resolution of microstructures and caused off‐target polymerization (Figure S1–S2, Supporting Information). Cell‐induced light scattering can be partially overcome by minimizing the refractive index difference between cells and their surrounding media. We minimized cell‐induced light scattering using refractive index matching (GelMA (5%) with iodixanol (40%), RI = 1.40) (Text S1–S3 and Figure S3–S4, Supporting Information), thereby allowing us to biofabricate cardiac tissues with high cell density bioinks.

**Figure 2:**
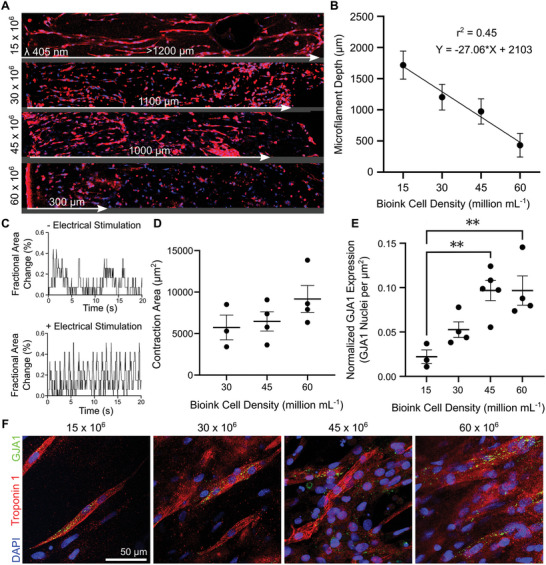
A high bioink cell density negatively impacts the hydrogel microfilament formation and cell alignment but improves contractility and gap junction protein expression in engineered tissues. A) Immunofluorescence mosaics of tissues (day 5) show how cell alignment reduces with increasing light penetration depth and cell concentration. The images were acquired between ≈20–120 µm tissue depth. B) Relationship between cell density of bioink and microfilament depth penetration (n  =  5, Pearson's correlation coefficient). C) Cardiac tissue contractility was measured during electrical stimulation (10 V, 1 Hz, 10% duty cycle) after 4–5 days in culture. Electrical stimulation led to the pacing of the contraction (spontaneous contraction (top), stimulated contraction (bottom)). D) Cardiac tissue contractility (measured as surface area change) at different biofabrication cell densities. E) Gap junction (GJA1) protein expression in fabricated tissues. GJA1‐expressing cells were counted in aligned regions of immunostained cardiac tissues (n = 5, one‐way ANOVA, ^**^ *p* < 0.01, and ^***^ *p* < 0.005). Here, GJA1 serves as an indicator of cell‐to‐cell signaling and connectivity. F) Immunostaining of cardiomyocytes and gap junctions in fabricated tissues.

Next, we measured cellular alignment in tissues fabricated at high cell density (15–60 × 10^6^ cells mL^−1^) (**Figure** **2**). We measured cellular alignment after 4‐5 days by analyzing images for directional local features computed from the gradient structure tensor (Figure 2A,B; Text S4 and Figure S5, Supporting Information).^[^ ^35^ ^]^ Our analysis of cardiomyocyte alignment shows that increasing cell density in the bioink causes a linear decrease in the depth of cellular alignment (Figure 2B; Figure S6; Pearson's correlation coefficient, r  =  −0.989). Therefore, our results align with the Beer‐Lambert Law, which relates cell concentration to absorbance.^[^ ^31^, ^33^, ^36^ ^]^ We also found that refractive index matching reduces, but does not eliminate, cell‐induced light scattering. Consequently, the filamented light‐induced cellular alignment depth is limited (≈1 mm) while fabricating tissues using high cell‐density bioinks.

### 2.2 Structural and Functional Characterization of Contractile Cardiac Tissues

We measured the structural and functional effect of biofabrication at high cell density by quantifying gap junction protein expression and tissue contraction. We kept the fabricated cardiac tissues in culture until the onset of synchronous, spontaneous tissue contraction (typically, 4–5 days). We electrically stimulated our cardiac tissues (10 V, 1 Hz, 10% duty cycle), and recorded tissue contractility (Figure 2C). The tissues which we biofabricated with 30, 45, and 60 × 10^6^ cells mL^−1^ contracted in response to electrical stimulation (Figure 2D). To assess structural changes in the tissues, we quantified GJA1 expression, an indicator of cell‐to‐cell connectivity and signaling (Figure 2E). GJA1 expression increased significantly in tissues biofabricated at 45 and 60 × 10^6^ cells mL^−1^, compared to 15 × 10^6^ cells mL^−1^. Immunostaining of cardiomyocytes showed elongation and cytoplasm striation (z‐lines) (Figure 2F; Figure S7, Supporting Information). GJA1 was distributed non‐physiologically across the cardiomyocyte sarcolemma versus the physiological localization of GJA1 in intercalated discs (connecting adjacent cardiomyocytes).^[^ ^37^ ^]^ Importantly, unlike native tissue, cells appear to elongate predominantly in the direction of alignment, with limited lateral spreading. We also observe a proportion of rounded cells, which may be embryonic cardiomyocytes, red blood cells, or quiescent or dead cardiomyocytes. Note that we observed rounded cells only in tissues that were fabricated with primary rat cardiomyocytes, not iPSC‐CMs. In summary, higher bioink concentrations (15–60 million cells mL^−1^) result in more contractile tissues, which express higher GJA1 levels; this, however, comes at a trade‐off with cellular alignment distance (>1200–300 µm).

### 2.3 Cell‐Induced Light Scattering Causes Anisotropic Tissue Remodelling and Stiffness

**Figure 3:**
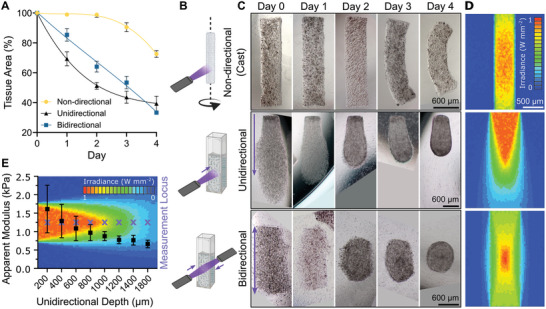
Cardiac tissue remodeling depends on the light irradiation mode. A) Change in tissue area (projected view) over time in the printed cardiac tissues. The cardiac tissues were fabricated (45 × 10^6^ cells mL^−1^ bioink density) with different irradiation modes (n = 3). The cardiac tissues were polymerized using casting/non‐directional light (360°), unidirectional, or bidirectional light. B) Schematic representation of different irradiation modes used during biofabrication. Purple arrows show the path length and orientation of the UV light. C) Microscopic images of hydrogel remodeling over 4 days in cell culture. D) Optical simulations showing cell‐induced light scattering with different irradiation modes in optically‐tuned bioink (45 × 10^6^ cells mL^−1^). E) Measurement of unidirectionally irradiated tissue stiffness along the light propagation path. The stiffness measurement locus is indicated in the unidirectional optical simulation.

We analyzed our tissues over 4 days to understand the influence of cell‐mediated light scattering on tissue remodeling (**Figure** **3**). We fabricated cardiac tissues using different irradiation modes (Figure 3A–C: non‐directional (360°), unidirectional, or bidirectional light irradiation), which influences irradiance distribution through the scattering media. The quantitative and qualitative data show that the tissue surface area decreased fastest in unidirectionally irradiated tissues, followed by bidirectionally irradiated tissues, and then non‐directionally irradiated tissues.

We investigated if differences in tissue remodeling rate were occurring due to differing irradiance between the different irradiation modes. Specifically, we developed an optical model of light scattering for each irradiation mode (Figure 3D; Text S5 and Figure S8, Supporting Information). Our model indicates that light irradiance in unidirectionally irradiated tissues varies the most, followed by bidirectionally irradiated and non‐directionally irradiated tissues, where irradiance is more homogeneous. We tested if these changes in irradiance affect hydrogel polymerization, by measuring tissue stiffness (Apparent Modulus) along the light propagation path in unidirectionally irradiated tissues (Figure 3E; Text S6, Supporting Information). Stiffness typically corresponds to crosslinking density, and crosslinking is caused by photopolymerization.^[^ ^38^ ^]^ Our measurements show that hydrogel stiffness decreases along the light propagation path (1.6 to 0.6 kPa), corresponding to the reducing irradiance shown in our optical stimulation. These results indicate that cell‐mediated light scattering causes differences in irradiance within the biofabricated tissues. These differences in irradiance result in different degrees of GelMA crosslinking, causing differences in tissue stiffness. These intra‐tissue variations in irradiance can be partially overcome by changing the irradiation method to non‐directional or bidirectional light.

### 2.4 Light Microstructures Can Direct Tissue Contractility and Increase GJA1 Expression

**Figure 4:**
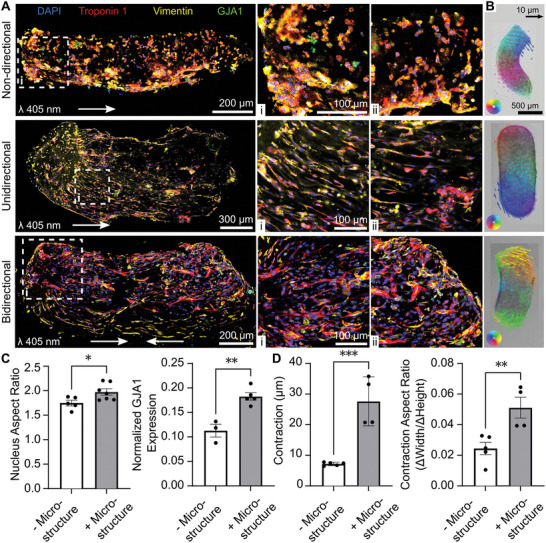
Structural and functional comparison of microstructured and unstructured (cast) cardiac tissues. A) Immunostaining mosaics of cardiac tissues that were biofabricated with non‐directional (cast), unidirectional, and bidirectional light irradiation and kept in culture for 4–5 days. Arrows indicate the approximate direction of light irradiation. Individual images show cell alignment in tissues at the start/end of the light propagation path. B) Optical flow (pixel‐wise velocity) of cardiac tissues during contraction. The contraction angle is colored using a Hue, Saturation, Value (HSV) wheel. Arrow sizes were scaled (25x) and filtered according to the magnitude of contraction by decreasing opacity for smaller contractions. C) Structural characterization of tissues with microstructures (bidirectional) and without microstructures (non‐directional, cast). Nuclear Aspect Ratio (n = 5, one‐way ANOVA); Normalized GJA1 Expression (GJA1 Nuclei per mm^2^) (n = 3, one‐way ANOVA) D) Tissue contractility characterization. Tissue contraction during electrical stimulation was determined by changes in the tissue width (n = 4, one‐way ANOVA); contraction aspect ratio was determined by measuring the difference in maximum/minimum aspect during electrical stimulation (n = 4, one‐way ANOVA, ^*^ *p* < 0.05, ^**^ *p* < 0.01, and ^***^ *p* < 0.005).

We evaluated how cellular alignment distance changes when using different irradiation modes (unidirectional versus bidirectional) for tissue biofabrication (**Figure** **4**). In tissues fabricated with non‐directional irradiation (cast/bulk polymerization), we observed no distinct cellular alignment due to the absence of microstructures (Figure 4A). Due to cell‐induced light scattering, we observed reduced cellular alignment in directionally irradiated tissues at increased light propagation depths (Figure S9, Supporting Information). However, we observed cellular alignment over larger distances in bidirectionally irradiated tissues, presumably due to the reduced light propagation depth. Together, these results indicate that bidirectional light irradiation can partially overcome the impact of cell‐induced light scattering on light‐induced cellular alignment.

Next, we measured how contractility changes in tissues irradiated with non‐directional, unidirectional, and bidirectional light. We used dense optical flow to compute changes in pixel velocity and contractility (Figure 4B).^[^ ^39^, ^40^ ^]^ Non‐directionally irradiated tissues are unstructured (no microfilaments or microchannels), while unidirectionally and bidirectionally irradiated tissues are microstructured. Cardiac tissues with microstructures contract distinctly since their axis of contractility is aligned with the axis of light irradiation. Furthermore, bidirectionally irradiated tissues contract symmetrically relative to unidirectionally irradiated tissues.

To evaluate the influence of light‐induced cell alignment on cardiac tissue structure and function, we analyzed cell nuclear aspect ratio, GJAI expression, and contractility. Specifically, we compared unstructured tissues (non‐directional) with microstructured tissues (bidirectional). The nucleus aspect ratio and GJA1 expression were higher in microstructured tissues (Figure 4C). Next, we assessed the effect of cell alignment on tissue contraction (Figure 4D); cellular alignment enhanced the unidirectional contractility (µm) of tissues by 4‐fold. Furthermore, microstructured tissues showed a 2‐fold increase in maximum contraction aspect ratio relative to unstructured controls. This result indicates that tissue contractility is directed along the direction of cellular alignment. We have also estimated the Specific Contractile Force (≈0.02 mN mm^−2^) of our tissues using the measured apparent modulus and linear contractility (Text S7, Supporting Information). In summary, these results show that FLight biofabrication can enhance gap‐junction protein expression (1.6x) and direct tissue contractility (3.8x).

### 2.5 Filamented Light Biofabrication Creates Multidirectional Cellular Alignment and Twisting Cardiac Tissue

**Figure 5:**
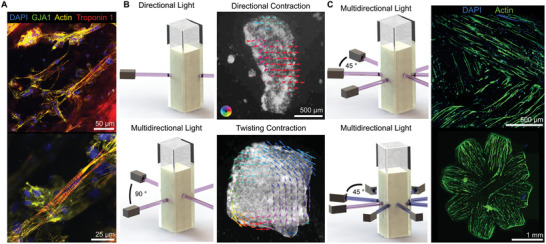
Multidirectional Light Biofabrication using Human iPSC‐CMs creates torsionally contracting tissues and multidirectional cellular alignment. A) Immunostaining of iPSC‐CM tissues after 12 days in culture. B) Biofabrication methodology to create directional or multilayered twisting iPSC‐CM cardiac muscle tissues, analyzed using optical flow (pixel‐wise velocity). C) Biofabrication methodology to create complex multidirectionally aligned cardiac tissues, including immunofluorescence images of a tri‐layered myocardium and single‐layer jellyfish‐structured tissue. Note that these two tissues were generated with rat cardiomyocytes and immunostained after 12 days in culture.

We fabricated tissues using iPSC‐CMs to test if FLight can be used to biofabricate human tissues (**Figure** **5**). Specifically, we used GelMA/matrigel hydrogels containing iPSC‐CMs at 45 × 10^6^ cells mL^−1^. The GelMA/matrigel tissues (ø 0.6 mm x 2 mm) were viable and contractile following incubation for ≈5 days (Figure S10, Supporting Information). Furthermore, cardiomyocytes in the microstructured tissues exhibited an elongated and striated morphology (Figure 5A), indicating sarcomeric organization and alignment. Next, we fabricated layered tissues (2 × 2 mm, 0.5 mm thickness) with multidirectional cellular alignments by projecting light in two layers at a 90° angle (Figure 5B; Figure S11, Supporting Information). As highlighted by our optical flow analysis, we observed a twisting contractility in the layered tissues (Videos [Link], [Link], [Link], [Link], Supporting Information). These results contrast to the directional contractility that was engineered with directional light projection. Next, we measured calcium (Ca^2+^) transient propagation to indicate cardiac impulse conduction in our tissues. We observed directional calcium transient propagation in our directional tissues and multidirectional transient propagation in our multidirectional tissues (Text S8, Videos S7 and S8, Supporting Information). While we observed a proportion of cells contracting synchronously and paced, we also observed cells contracting asynchronously. This observation may suggest that a subset of cells lack cell‐to‐cell contact. Finally, we compared tissue impulse conduction and contraction direction; we observed an overlap in the directionality and therefore agreement between the two results (Figure S12, Supporting Information).

We further expanded our multidirectional alignment concept by fabricating a myocardium and jellyfish‐structured tissue (ø 8 mm, 0.5–0.6 mm thickness). Both of these tissues were fabricated using rat cardiomyocytes. Specifically, to form the myocardium‐like tissue, we projected light at 45° angles in three distinct layers (Figure 5C, top). To fabricate the jellyfish‐structured tissue, we projected light in a single layer at 45° angles (Figure 5C, bottom). However, due to the absence of a strain‐limiting layer (e.g., PDMS), the jellyfish‐structured tissue did not contract directionally and, therefore, did not swim. These results show how FLight biofabrication can be applied to engineer complex cellular alignment patterns and influence tissue contractility.

## 3 Discussion

We have shown that multidirectional FLight can biofabricate contractile 3D cardiac tissues with controlled cellular alignment and contractility. Microstructured cardiac tissues were biofabricated using rat cardiomyocytes and human iPSC‐CMs at high cellular density. The resulting tissues exhibited an enhanced, directed contractility with improved cell‐to‐cell connectivity (gap‐junction protein expression), relative to unstructured control tissues. Even with optical tuning of our bioink, high cell bioink densities adversely affected the formation of the hydrogel microstructures. This cell‐induced light attenuation causes anisotropic tissue contraction and remodeling in the fabricated tissues. These challenges can be partially overcome using multidirectional light projection, which increases the alignment depth, and improves tissue homogeneity. Furthermore, this approach can be used to biofabricate cardiac tissues with complex, multidirectional cellular alignment and biomimetic contractility. Specifically, we have fabricated layered tissues containing two alignment directions that contract in a twisting motion. This contractility is similar to the torsional motion of the heart myocardium. Finally, by fabricating a tri‐layered myocardium and jellyfish‐structured tissue, we have shown how multidirectional FLight can generate relevant tissue structures for regenerative medicine and biohybrid robotics.

Our work also has limitations. The observed differences in the intra‐ and inter‐tissue stiffness may affect cell metabolism and force generation, cell mobility and remodeling, and cell maturation within and between our tissues.^[^ ^41^ ^]^ Next, we compared tissue functionality (contractility, GJA1 expression) between microstructured and unstructured tissues. Future work should characterize performance metrics more comprehensively, for example, tissue force generation, calcium handling and propagation speed, and cell maturation. Furthermore, these metrics could be benchmarked against tissue generated with other cell alignment methodologies. Our estimated value for Specific Contractile Force (≈0.02 mN mm^−2^) is lower but comparable to other cardiac tissues fabricated with rat cardiomyocytes (0.1–0.6 mN mm^−2^).^[^ ^42^ ^]^ Regarding cell maturation, it is important to note that FLight improves cell‐to‐cell interactions in the direction of alignment but hinders it in the lateral dimension. Therefore, the FLight hydrogel microstructure may affect physiological cell maturation and tissue formation. Next, FLight‐aligned tissues must be selected according to the desired application and appropriate timeframe. The induced cellular alignment requires a hydrogel microstructure, which will eventually be remodeled, potentially leading to some loss of alignment. Here, we have characterized our tissues after a maximum period of 18 days and with up to three cell alignment layers. Future work could explore how FLight‐induced cellular alignment is maintained over longer periods and how this method can be combined with complementary cell maturation techniques to fabricate tissues with durable multi‐layered alignment. Finally, overcoming cell‐induced light scattering is key to fabricating non‐proliferative cells/tissue types, such as cardiac and neuronal tissues. Here, improved polymerization chemistries, optical techniques, or the use of proliferative, pluripotent cells could extend the applicability of this technology.^[^ ^31^, ^32^, ^41^ ^]^

Our approach here has several advantages relative to other state‐of‐the‐art biofabrication techniques for generating aligned and contractile cardiac tissues. Firstly, FLight cell alignment can rapidly create multiple tissues in parallel or large, complex tissue geometries (<30 s). Furthermore, unlike other alignment techniques, FLight can induce cellular alignment without prior micropatterning or molding,^[^ ^12^ ^]^ preparation of building blocks,^[^ ^8^ ^]^ or additive elements.^[^ ^9^, ^18^ ^]^ Finally, FLight is one of only a few techniques (Table S1, Supporting Information) capable of in situ generation of multidirectional cellular alignments in cell‐laden hydrogels; this characteristic is essential for fabricating 3D tissues homogeneously distributed with cells. Further development could enable FLight to fabricate large, centimeter‐scale tissues with increasingly complex 3D alignment patterns and contractility, such as torsionally contracting helical tissues.^[^ ^24^ ^]^ Such developments would allow the full application of FLight‐fabricated tissues in areas such as in vitro tissue models, regenerative medicine, and biohybrid robotics.

## 4 Conclusion

We have shown that filamented light biofabrication can be used to fabricate high‐cell‐density, aligned, and directionally contractile cardiac tissues. Furthermore, by projecting light multidirectionally, tissues can be fabricated with multidirectional cellular alignment and a tunable contractility (e.g., linear versus twisting). Therefore, we envision that multidirectional FLight will be highly relevant in muscle tissue engineering or biohybrid robotics. Specifically, FLight can be used to rapidly and scalably induce cellular alignment in situ without prior substrate patterning or using additive elements. These factors make FLight‐induced cellular alignment highly accessible for inducing complex cellular alignment in 3D cell‐laden hydrogels.

## 5 Experimental Section

### Cardiac Cell Isolation and Culture

Unless stated, all chemicals were purchased from Sigma‐Aldrich, and cell culture reagents from Thermofischer (Gibco). Tissues were obtained from the disposed cadavers of animals that were sacrificed for another research group. Animal handling procedures were performed under the Canton of Zurich guidelines for animal experimentation. Embryonic rat ventricular myocytes (E18.5) were purified from the embryos of a Sprague Dawley Rat. Rats were anesthetized using 5% isoflurane and decapitated. Rat cardiomyocytes were dissociated using a Neonatal Heart Dissociation Kit (gentleMACS, Miltenyi Biotec) and isolated using a Neonatal Cardiomyocyte Isolation Kit (gentleMACS, Miltenyi Biotec) according to the manufacturer's instructions. Rat cardiomyocytes were cultured with DMEM/F12 medium supplemented with L‐glutamine (1% v/v), sodium pyruvate (1% v/v), non‐essential amino acids (1% v/v), Primocin (0.2% v/v) (InvivoGen), horse serum (5% v/v) and fetal bovine serum (FBS) (10% v/v) at 37 °C with 5% CO_2_.^[^ ^43^ ^]^ Human iPSCs were donated by a 33‐year‐old healthy female. iPSCs were reprogrammed by the CytoTune‐iPS Sendai Reprogramming Kit (ThermoFisher) and cultured in StemMACS iPSC‐Brew Medium (Miltenyi Biotec). They were differentiated into iPSC‐CMs at 80% confluence using StemMACS CardioDiff Kit XF (Miltenyi Biotec) following the manufacturer's instructions. On day 10 of differentiation, the cells were detached and used for biofabrication. The fabricated tissues were cultured in RPMI 1640 supplemented with B27 (2% v/v), FBS (10% v/v), Rock Inhibitor (2 µM) (StemMACS Y27632, Miltenyi Biotec), and Primocin (0.2% v/v) for 2–3 days and subsequently in RPMI 1640, B27 (2% v/v), and Primocin (0.2% v/v).

### Synthesis of GelMA and Photoink Preparation

GelMA (40% DoF) was synthesized as previously described.^[^ ^44^ ^]^ After the reaction, the mixture was dialyzed against deionized water for 4 days at 40 °C to remove unreacted monomers, with subsequent freeze drying. GelMA (5%, w/v) was dissolved in PBS with Lithium phenyl‐ 2,4,6‐trimethylbenzoylphosphinate (0.1%, w/v) and Iodixanol (30%, w/v) (OptiPrep Density Gradient Medium, StemCell Technologies). When using iPSC‐CMs, Matrigel Matrix (0.4 mg mL^−1^, protein concentration) (Corning) was added. The mixture was resuspended with cardiac cells at a predetermined concentration to form the printable bioink.

### Biofabrication

Bioink was added to sterile UV‐transparent cuvettes (UVette, Eppendorf) and gelated by incubation at 4 °C. The bioink was irradiated with UV light using a volumetric bioprinter (Tomolite, Readily3D) in projection mode (no rotation). An image containing a circle (ø 500 µm) was uploaded to the DMD and projected onto the sample. The printing dose (irradiation time) was calibrated according to each batch of GelMA: Typically, a dose of 500–800 mJ cm^−2^ was used (Text S3 Supporting Information). After printing, unpolymerized GelMA was removed by washing with warm PBS (37 °C). Tissues were then transferred to cardiomyocyte culture media. Tissues fabricated with unidirectional polymerization were only irradiated with UV light from one orientation. Bidirectional tissues were irradiated from one side with half the final UV dose, rotated 180°, and irradiated with the remaining dose. Non‐directional tissues were polymerized by adding bioink to PTFE tubing (⌀ 500 µm) and rotated (72 rpm) with UV irradiation. For non‐directional irradiation experiments, a dose of ≈1.5x (1000–1300 mJ cm^−2^) was used. For multidirectional projection experiments, images were projected from different angles (twisting tissue: (90°, 2 × 0.25 mm, 2 layers), jellyfish‐structured tissue: 45°, 1 mm x 0.2 mm, one layer, myocardium‐like tissue: 60°, 0.1 mm x 10 mm, three layers). Bioink was added to borosilicate glass vials (5 mm O.D) to fabricate the jellyfish and myocardium‐like tissues at a concentration of 15 × 10^6^ cells mL^−1^.

### Optical Microscopy and Electrical Stimulation

Tissues were imaged using an optical microscope (Olympus CKX41). After 4–5 days of cell culture, tissues were electrically stimulated (10 Volts, 1 Hz, 10% duty cycle) using parallel graphite electrodes (2 cm spacing), submerged in the cell culture media, connected to a function generator via platinum wiring. All electrical stimulation was performed in warm (37 °C) cardiomyocyte culture media.

### Contraction Analysis

Tissue contractility was measured after 4–5 days of cell culture. Videos were recorded using a camera (Olympus SC50) at a frame rate of 30 fps and processed using Fiji (ImageJ).^[^ ^45^ ^]^ The contraction area (µm^2^, Figure 2) was measured by thresholding images and performing particle analysis across the entire image stack. Change in contraction area was then determined by subtracting the minimum recorded area from the maximum recorded area within a 1 s interval. Uniaxial contraction (µm, Figure 4D) was determined by measuring the change in max‐min width (bounding box, Fiji calculated). Width corresponded to the largest dimension of the tissues. The aspect ratio of the contraction was determined by averaging the change in the calculated aspect ratio (AR, Analyze Particles, Fiji) across the entire image stack. Note that the change between contraction area (µm^2^) and uniaxial contraction (µm) was made due to the relative increase in background noise while analyzing the contractility of unstructured tissues. All data analysis was performed on a minimum of 20 s recorded contractility.

### Alignment Analysis

Optical microscopy images were rotated horizontally and processed using OrientationJ (EPFL).^[^ ^35^ ^]^ Cellular alignment depth was then manually measured using Fiji. Optical microscopy images typically focus only on the surface of tissues. Alignment depth was defined as the point at which a repeating, consistent alignment pattern could no longer be identified.

### Gap Junction Analysis

Gap junction expression was determined by counting cell nuclei near gap junctions. Cells expressing GJA1 but not interfacing with another cell were not counted. GJA1 expression was normalized to the number of counted cell nuclei. Gap junctions were counted across entire regions of tissues. The nuclear aspect ratio was determined using the blue (DAPI) channel of fluorescence imaging. Nuclei in the aligned regions of tissues were measured by thresholding the image, performing particle analysis, and averaging the measured aspect ratio.

### Optical Flow Analysis

Previous work has shown how non‐invasive optical flow analysis can provide information on the contraction of cells and tissues during video post‐processing.^[^ ^39^ ^]^ An open‐source (OpenCV) optical flow algorithm was used to visualize tissue contraction using Gunnar Farneback's algorithm.^[^ ^46^, ^47^ ^]^ This algorithm provides a dense pixel‐wise velocity measure between different frames of contraction. The contraction direction was colored using a Hue, Saturation, Value (HSV) wheel. Arrow size and opacity were scaled with the magnitude of contraction. For visibility, arrow length was scaled by a factor of 25, and only a subset of arrows were shown by uniformly sampling 1% of the pixels.

### Immunofluorescence Staining

Cardiac tissues were washed in PBS and fixed in 4% paraformaldehyde for 30 min at 23 °C. The tissues were permeabilized with Triton‐X 100 (0.1%) in PBS for 30 min before blocking with donkey serum (5% v/v) in PBS for 2 h. Tissues were then incubated with primary antibodies (1/100, Anti‐Cardiac Troponin I antibody (ab47003), Anti‐Vimentin antibody (ab24525), Anti‐Connexin 43/GJA1 antibody(ab219493), Abcam) in donkey serum (5%) overnight at 4 °C. Next, the tissues were washed 3 times with Tween‐20 (0.1%, v/v), and incubated with secondary antibody (1/1000 Donkey Anti‐Rabbit IgG H&L (Alexa Fluor 647) (ab150075), Abcam), (Alexa Fluor 594 AffiniPure Donkey Anti‐Chicken IgY (IgG) (H+L), Jackson), (Donkey Anti‐Goat IgG H&L (Alexa Fluor 488) preadsorbed (ab150133), Abcam), in donkey serum (5%) at 24 °C, for 2 h. Alternatively, tissues were incubated with an actin stain (ActinGreen 488 or ActinRed 555, ReadyProbes, Thermo Fischer Scientific) for 2 h. Cell nuclei were stained in DAPI (300 µm) for 30 min at 23 °C. Samples were washed in Tween‐20 (0.1%, v/v), and then mounted in mounting media (ProLong Diamond Antifade Mountant, Thermo Fischer Scientific). A cover slip was gently placed on top of the sample to achieve a uniform imaging depth. Imaging was performed on a Leica SP8‐AOBS‐CARS Confocal Microscope. Tissues were imaged at the surface of the tissue. Confocal mosaics were acquired with the mosaic function and merged using LAS X (Leica).

### Atomic Force Microscopy

AFM nanoindentation was performed using a Flex‐Bio AFM (Nanosurf, Switzerland) on tissues at Day 0. The tissue was indented using a colloidal probe consisting of a soft cantilever with a nominal spring constant (k = 0.1 N m^−1^) and a borosilicate glass bead (⌀ 10 µm) attached to the cantilever tip (CP‐qp‐CONT‐BSG‐B‐5; Nanosensors). The spring constant was obtained using the Sader method. The deflection‐displacement was recorded during the indentation of a glass slide to obtain the deflection sensitivity. Measurements were performed starting at ≈100 µm from the beginning of the tissues and at 200 µm intervals thereafter. The deflection and displacement of the cantilever were measured to produce a force‐displacement curve for each location. Typically, eight 50 µm^2^ regions (5 × 5 measurement grid) were indented at 200 µm intervals within the tissue. The apparent modulus (*Is*) was obtained from force‐displacement curves by fitting them to the Hertz contact model for a sphere indenting a semi‐infinite half‐space. Force‐displacement curves without a clear contact point were discarded.

### Optical Simulation

Optical simulations were performed using a FRED Optical Engineering Software (Photon Engineering, USA) using a Mie scattering model. All simulations consider the interaction of 405 nm UV (405 nm) light with an optically tuned, cell‐laden bioink (GelMA (5%), Iodixanol (30%), with 45 × 10^6^ cells mL^−1^. Parameters were either calculated (mean free path) or measured (particle size, beam radius, particle and media index, beam divergence). Simulations were developed for the different printing modalities by considering the orientation of UV light (unidirectional: light from one direction; bidirectional: light from both directions; non‐directional: light from a cylindrical source). A detector was then placed through the cross‐section of the beam for results generation.

### Statistical Analysis

Data was pre‐processed according to the experiment: tissue alignment analysis was performed on tissues in which cell outlines were visible (optical microscopy); contractility analysis was performed on selected videos of contractile tissues with minimal noise and maximum image contrast; GJA1 analysis was performed on several (≈¼ of total tissue area) high contrast images from tissue confocal mosaics, and was normalized to area; nuclear aspect ratio analysis was performed on selected high‐contrast images in the aligned regions of tissues. Plotting and Statistical analysis were performed using GraphPad Prism (x64, v. 9.5.1). Data is presented as Mean ± SEM (Standard Error of the Mean). One‐way ANOVA (unpaired, gaussian distribution, using a Tukey test) was performed in Figures 2A and 3C. T‐test (unpaired, gaussian distribution, two‐tailed) was performed in Figure 4C,D. For all tests, the alpha was set to 0.05. Differences between the two experimental groups were judged to have statistical significance at ^*^ *p* < 0.05, ^**^ *p* < 0.01, and ^***^ *p* < 0.005; groups with no significant difference are not indicated.

## Conflict of Interest

The authors declare no conflict of interest.

## Author Contributions

L.J. designed the study, performed experiments and data analysis, created figures, wrote the manuscript, and revised it. M.F. supported experiment planning, data analysis, and figure generation and revised the manuscript. M.M. performed optical flow characterization and generated figures. A.B. supported the experiments and data analysis and revised the manuscript. O.Y. supported the experimental planning and revised the manuscript. G.A. conceived the initial experiments, supported the research with protocols and knowledge, and performed preliminary experiments. M.N. performed AFM measurements and data analysis. S.F. coordinated the animal sacrifice and performed animal surgery. M.G. provided the iPSC‐CMs. E.T. conceived the initial experiments and supported the research with protocols and knowledge. R.K. acquired the funding, conceived the study, supervised the research, wrote the manuscript, generated figures, and revised the manuscript.

## Supporting information

Supporting Information

Supplemental Video 1

Supplemental Video 2

Supplemental Video 3

Supplemental Video 4

Supplemental Video 5

Supplemental Video 6

Supplemental Video 7

Supplemental Video 8

Supplemental Response Video 1

## Data Availability

The data that support the findings of this study are available from the corresponding author upon reasonable request.

## References

[1] P. P. Sengupta , A. J. Tajik , K. Chandrasekaran , B. K. Khandheria , JACC Cardiovasc. Imaging. 2008, 1, 366. 19356451 10.1016/j.jcmg.2008.02.006

[2] X. Yang , L. Pabon , C. E. Murry , Circ. Res. 2014, 114, 511. 24481842 10.1161/CIRCRESAHA.114.300558 PMC3955370

[3] N. T. Feric , M. Radisic , Adv. Drug. Delivery. Rev. 2016, 96, 110. 10.1016/j.addr.2015.04.019 PMC4635107 25956564

[4] N. T. Feric , I. Pallotta , R. Singh , D. R. Bogdanowicz , M. M. Gustilo , K. W. Chaudhary , R. N. Willette , T. P. Chendrimada , X. Xu , M. P. Graziano , R. Aschar‐Sobbi , Toxicol. Sci. 2019, 172, 89. 31385592 10.1093/toxsci/kfz168 PMC6813749

[5] I. Mannhardt , K. Breckwoldt , D. Letuffe‐Brenière , S. Schaaf , H. Schulz , C. Neuber , A. Benzin , T. Werner , A. Eder , T. Schulze , B. Klampe , T. Christ , M. N. Hirt , N. Huebner , A. Moretti , T. Eschenhagen , A. Hansen , Stem. Cell. Rep. 2016, 7, 29. 10.1016/j.stemcr.2016.04.011 PMC4944531 27211213

[6] A. Lee , A. R. Hudson , D. J. Shiwarski , J. W. Tashman , T. J. Hinton , S. Yerneni , J. M. Bliley , P. G. Campbell , A. W. Feinberg , Science 2019, 365, 482. 31371612 10.1126/science.aav9051

[7] M. E. Kupfer , W.‐H. Lin , V. Ravikumar , K. Qiu , L. Wang , L. Gao , D. B. Bhuiyan , M. Lenz , J. Ai , R. R. Mahutga , D. Townsend , J. Zhang , M. C. McAlpine , E. G. Tolkacheva , B. M. Ogle , Circ. Res. 2020, 127, 207. 32228120 10.1161/CIRCRESAHA.119.316155 PMC8210857

[8] J. Ahrens , S. Uzel , M. Skylar‐Scott , M. Mata , A. Lu , K. Kroll , J. A. Lewis , Adv. Mater. 2022, 34, 2200217. 10.1002/adma.202200217 35451188

[9] S. Choi , K. Y. Lee , S. L. Kim , L. A. MacQueen , H. Chang , J. F. Zimmerman , Q. Jin , M. M. Peters , H. A. M. Ardoña , X. Liu , A.‐C. Heiler , R. Gabardi , C. Richardson , W. T. Pu , A. R. Bausch , K. K. Parker , Nat. Mater. 2023, 22, 1039. 37500957 10.1038/s41563-023-01611-3 PMC10686196

[10] W. Bian , C. P. Jackman , N. Bursac , Biofabrication 2014, 6, 024109. 24717534 10.1088/1758-5082/6/2/024109 PMC4040155

[11] H. Sun , J. Zhou , Z. Huang , L. Qu , N. Lin , C. Liang , R. Dai , L. Tang , F. Tian , Int. J. Nanomed. 2017, 12, 3109. 10.2147/IJN.S128030 PMC5399986 28450785

[12] J. C. Nawroth , H. Lee , A. W. Feinberg , C. M. Ripplinger , M. L. McCain , A. Grosberg , J. O. Dabiri , K. K. Parker , Nat. Biotechnol. 2012, 30, 792. 22820316 10.1038/nbt.2269 PMC4026938

[13] D. Zhang , I. Y. Shadrin , J. Lam , H.‐Q. Xian , H. R. Snodgrass , N. Bursac , Biomaterials 2013, 34, 5813. 23642535 10.1016/j.biomaterials.2013.04.026 PMC3660435

[14] M. Tiburcy , J. E. Hudson , P. Balfanz , S. Schlick , T. Meyer , M.‐L. C. Liao , E. Levent , F. Raad , S. Zeidler , E. Wingender , J. Riegler , M. Wang , J. D. Gold , I. Kehat , E. Wettwer , U. Ravens , P. Dierickx , L. W. van Laake , M. J. Goumans , S. Khadjeh , K. Toischer , G. Hasenfuss , L. A. Couture , A. Unger , W. A. Linke , T. Araki , B. Neel , G. Keller , L. Gepstein , J. C. Wu , et al., Circulation 2017, 135, 1832. 28167635 10.1161/CIRCULATIONAHA.116.024145 PMC5501412

[15] N. Strash , S. DeLuca , G. L. Janer Carattini , Y. Chen , T. Wu , A. Helfer , J. Scherba , I. Wang , M. Jain , R. Naseri , N. Bursac , Sci. Adv. 2024, 10, eadh2598. 38266090 10.1126/sciadv.adh2598 PMC10807800

[16] N. Tandon , C. Cannizzaro , P.‐H. G. Chao , R. Maidhof , A. Marsano , H. T. H. Au , M. Radisic , G. Vunjak‐Novakovic , Nat. Protoc. 2009, 4, 155. 19180087 10.1038/nprot.2008.183 PMC2775058

[17] S. S. Nunes , J. W. Miklas , J. Liu , R. Aschar‐Sobbi , Y. Xiao , B. Zhang , J. Jiang , S. Massé , M. Gagliardi , A. Hsieh , N. Thavandiran , M. A. Laflamme , K. Nanthakumar , G. J. Gross , P. H. Backx , G. Keller , M. Radisic , Nat. Methods. 2013, 10, 781. 23793239 10.1038/nmeth.2524 PMC4071061

[18] C. Licht , J. C. Rose , A. O. Anarkoli , D. Blondel , M. Roccio , T. Haraszti , D. B. Gehlen , J. A. Hubbell , M. P. Lutolf , L. De Laporte , Biomacromolecules 2019, 20, 4075. 31614080 10.1021/acs.biomac.9b00891

[19] S. Richard , A. K. A. Silva , G. Mary , H. Ragot , J. E. Perez , C. Ménager , F. Gazeau , I. Boucenna , O. Agbulut , C. Wilhelm , ACS Appl. Bio. Mater. 2020, 3, 6802. 10.1021/acsabm.0c00754 35019343

[20] D. V. Deshmukh , P. Reichert , J. Zvick , C. Labouesse , V. Künzli , O. Dudaryeva , O. Bar‐Nur , M. W. Tibbitt , J. Dual , Adv. Funct. Mater. 2022, 32, 2113038.

[21] J. P. K. Armstrong , J. L. Puetzer , A. Serio , A. G. Guex , M. Kapnisi , A. Breant , Y. Zong , V. Assal , S. C. Skaalure , O. King , T. Murty , C. Meinert , A. C. Franklin , P. G. Bassindale , M. K. Nichols , C. M. Terracciano , D. W. Hutmacher , B. W. Drinkwater , T. J. Klein , A. W. Perriman , M. M. Stevens , Adv. Mater. 2018, 30, 1802649. 30277617 10.1002/adma.201802649 PMC6386124

[22] V. Serpooshan , P. Chen , H. Wu , S. Lee , A. Sharma , D. A. Hu , S. Venkatraman , A. V. Ganesan , O. B. Usta , M. Yarmush , F. Yang , J. C. Wu , U. Demirci , S. M. Wu , Biomaterials 2017, 131, 47. 28376365 10.1016/j.biomaterials.2017.03.037 PMC5446052

[23] T. U. Esser , A. Anspach , K. A. Muenzebrock , D. Kah , S. Schrüfer , J. Schenk , K. G. Heinze , D. W. Schubert , B. Fabry , F. B. Engel , Adv. Mater. 2023, 35, 2305911. 10.1002/adma.202305911 37655652

[24] H. Chang , Q. Liu , J. F. Zimmerman , K. Y. Lee , Q. Jin , M. M. Peters , M. Rosnach , S. Choi , S. L. Kim , H. A. M. Ardoña , L. A. MacQueen , C. O. Chantre , S. E. Motta , E. M. Cordoves , K. K. Parker , Science 2022, 377, 180. 35857545 10.1126/science.abl6395 PMC10077766

[25] K. L. Miller , Y. Xiang , C. Yu , J. Pustelnik , J. Wu , X. Ma , T. Matsui , K. Imahashi , S. Chen , Organs‐on‐a‐Chip 2021, 3, 100007.

[26] M. Radisic , M. Euloth , L. Yang , R. Langer , L. E. Freed , G. Vunjak‐Novakovic , Biotechnol. Bioeng. 2003, 82, 403. 12632397 10.1002/bit.10594

[27] O. Bergmann , S. Zdunek , A. Felker , M. Salehpour , K. Alkass , S. Bernard , S. L. Sjostrom , M. Szewczykowska , T. Jackowska , C. Dos Remedios , T. Malm , M. Andrä , R. Jashari , J. R. Nyengaard , G. Possnert , S. Jovinge , H. Druid , J. Frisén , Cell 2015, 161, 1566. 26073943 10.1016/j.cell.2015.05.026

[28] I. B. Burgess , W. E. Shimmell , K. Saravanamuttu , J. Am. Chem. Soc. 2007, 129, 4738. 17378567 10.1021/ja068967b

[29] D. Kip , M. Soljacic , M. Segev , E. Eugenieva , D. N. Christodoulides , Science 2000, 290, 495. 11039925 10.1126/science.290.5491.495

[30] H. Liu , P. Chansoria , P. Delrot , E. Angelidakis , R. Rizzo , D. Rütsche , L. A. Applegate , D. Loterie , M. Zenobi‐Wong , Adv. Mater. 2022, 34, 2204301. 10.1002/adma.202204301 36095325

[31] P. N. Bernal , M. Bouwmeester , J. Madrid‐Wolff , M. Falandt , S. Florczak , N. G. Rodriguez , Y. Li , G. Größbacher , R.‐A. Samsom , M. van Wolferen , L. van der Laan , P. Delrot , D. Loterie , J. Malda , C. Moser , B. Spee , R. Levato , Adv. Mater. 2022, 34, 2110054. 10.1002/adma.202110054 35166410

[32] J. Madrid‐Wolff , A. Boniface , D. Loterie , P. Delrot , C. Moser , Adv. Sci. 2022, 9, 2105144. 10.1002/advs.202105144 PMC9353445 35585671

[33] S. You , Y. Xiang , H. H. Hwang , D. B. Berry , W. Kiratitanaporn , J. Guan , E. Yao , M. Tang , Z. Zhong , X. Ma , D. Wangpraseurt , Y. Sun , T. Lu , S. Chen , Sci. Adv. 2023, 9, eade7923. 36812321 10.1126/sciadv.ade7923 PMC9946358

[34] A. M. Nicks , S. R. Holman , A. Y. Chan , M. Tsang , P. E. Young , D. T. Humphreys , N. Naqvi , A. Husain , M. Li , N. J. Smith , S. E. Iismaa , R. M. Graham , J. Mol. Cell. Cardiol. 2022, 170, 47. 35644482 10.1016/j.yjmcc.2022.05.012

[35] Z. Püspöki , M. Storath , D. Sage , M. Unser , in Focus Bio‐Image Informatics (Eds: W. H. De Vos , S. Munck , J.‐P. Timerman ), Springer International Publishing, New York 2016, pp. 69–93.

[36] D. F. Swinehart , J. Chem. Educ. 1962, 39, 333.

[37] S. Funakoshi , I. Fernandes , O. Mastikhina , D. Wilkinson , T. Tran , W. Dhahri , A. Mazine , D. Yang , B. Burnett , J. Lee , S. Protze , G. D. Bader , S. S. Nunes , M. Laflamme , G. Keller , Nat. Commun. 2021, 12, 3155. 34039977 10.1038/s41467-021-23329-z PMC8155185

[38] I. Pepelanova , K. Kruppa , T. Scheper , A. Lavrentieva , Bioengineering 2018, 5, 55. 30022000 10.3390/bioengineering5030055 PMC6165498

[39] D. Zoccolan , A. Giachetti , V. Torre , J. Neurosci. Methods. 2001, 110, 65. 11564526 10.1016/s0165-0270(01)00418-6

[40] A. Czirok , D. G. Isai , E. Kosa , S. Rajasingh , W. Kinsey , Z. Neufeld , J. Rajasingh , Sci. Rep. 2017, 7, 10404. 28871207 10.1038/s41598-017-10094-7 PMC5583397

[41] C. C. Cook , E. J. Fong , J. J. Schwartz , D. H. Porcincula , A. C. Kaczmarek , J. S. Oakdale , B. D. Moran , K. M. Champley , C. M. Rackson , A. Muralidharan , R. R. McLeod , M. Shusteff , Adv. Mater. 2020, 32, 2003376. 10.1002/adma.202003376 33002275

[42] W. Dou , M. Malhi , Q. Zhao , L. Wang , Z. Huang , J. Law , N. Liu , C. A. Simmons , J. T. Maynes , Y. Sun , Microsyst. Nanoeng. 2022, 8, 26. 35299653 10.1038/s41378-021-00344-0 PMC8882466

[43] A. Aharonov , A. Shakked , K. B. Umansky , A. Savidor , A. Genzelinakh , D. Kain , D. Lendengolts , O.‐Y. Revach , Y. Morikawa , J. Dong , Y. Levin , B. Geiger , J. F. Martin , E. Tzahor , Nat. Cell. Biol. 2020, 22, 1346. 33046882 10.1038/s41556-020-00588-4

[44] B. Kessel , M. Lee , A. Bonato , Y. Tinguely , E. Tosoratti , M. Zenobi‐Wong , Adv. Sci. 2020, 7, 2001419. 10.1002/advs.202001419 PMC7509724 32999847

[45] J. Schindelin , I. Arganda‐Carreras , E. Frise , V. Kaynig , M. Longair , T. Pietzsch , S. Preibisch , C. Rueden , S. Saalfeld , B. Schmid , J.‐Y. Tinevez , D. J. White , V. Hartenstein , K. Eliceiri , P. Tomancak , A. Cardona , Nat. Methods. 2012, 9, 676. 22743772 10.1038/nmeth.2019 PMC3855844

[46] B. K. P. Horn , B. G. Schunck , Artif. Intell. 1981, 17, 185.

[47] B. D. Lucas , T. Kanade , *Proc. 7th Intl. Joint Conf. Artif. Intell*. Vancouver, BC August 1981.

