# Supplementary material for: Multidirectional Filamented Light Biofabrication Creates Aligned and Contractile Cardiac Tissues
Source: Adv Sci (Weinh). 2024 Oct 7;11(47):2404509. doi: 10.1002/advs.202404509 (PMC11653608; doi:10.1002/advs.202404509)
Supplement: Supplementary file 1 — Supporting Information [file ADVS-11-2404509-s003.pdf]

ADVANCED SCIENCE
Open Access

Supporting Information

for *Adv. Sci.*, DOI 10.1002/advs.202404509

Multidirectional Filamented Light Biofabrication Creates Aligned and Contractile Cardiac Tissues

*Lewis S. Jones, Miriam Filippi, Mike Yan Michelis, Aiste Balciunaite, Oncay Yasa, Gal Aviel, Maria Narciso, Susanne Freedrich, Melanie Generali, Eldad Tzahor and Robert K. Katzschmann**

Multidirectional Filamented Light Biofabrication Creates Aligned and Contractile Cardiac Tissues

*Lewis S. Jones[1], Miriam Filippi[1], Mike Yan Michelis[1], Aiste Balciunaite[1], Oncay Yasa[1], Gal Aviel[2], Maria Narciso[3,4], Susanne Freedrich[5], Melanie Generali[6], Eldad Tzahor[2], Robert K. Katzschmann[1]**

[1] Soft Robotics Laboratory, ETH Zurich, Tannenstrasse 3, Zurich, 8092, Switzerland
[2] Department of Molecular Cell Biology, Weizmann Institute of Science, Rehovot, 76100, Israel
[3] Swiss Federal Laboratories for Materials Science and Technology (EMPA), Dubendorf, 8600, Switzerland
[4] Experimental Continuum Mechanics, ETH Zurich, Leonhardstrasse 21, Zurich, 8092, Switzerland
[5] ETH Phenomics Center, ETH Zurich, Otto-Stern-Weg 7, Zurich, 8093, Switzerland
[6] Institute for Regenerative Medicine (IREM), University of Zurich, Schlieren, 8952, Switzerland

* Corresponding author's email: rkk@ethz.ch

This PDF includes Figures S1-S12, Table S1, and Supplementary Materials and Methods.

Other Supplementary Materials for this manuscript include:

- Video S1 - Immunofluorescence (GJA1 and autofluorescence) Z-stack showing microfilaments in engineered cardiac tissue (bioprinted using 45 × 10^6 cells/mL)
- Video S2 - Immunofluorescence (Troponin I) Z-stack showing cell alignment (bioprinted using 45 × 10^6 cells mL^{-1})
- Video S3 - Extended contractility recording of a directional tissue fabricated with iPSC-CMs at 45 × 10^6
- Video S4 - Extended contractility recording of a twisting cardiac tissue, fabricated with iPSC-CMs at 45 × 10^6 cells mL^{-1}
- Video S5 - Optical flow analysis on a directional cardiac tissue
- Video S6 - Optical flow analysis on a twisting cardiac tissue
- Video S7 - Calcium Imaging on a directional cardiac tissue
- Video S8 - Calcium Imaging on a twisting cardiac tissue

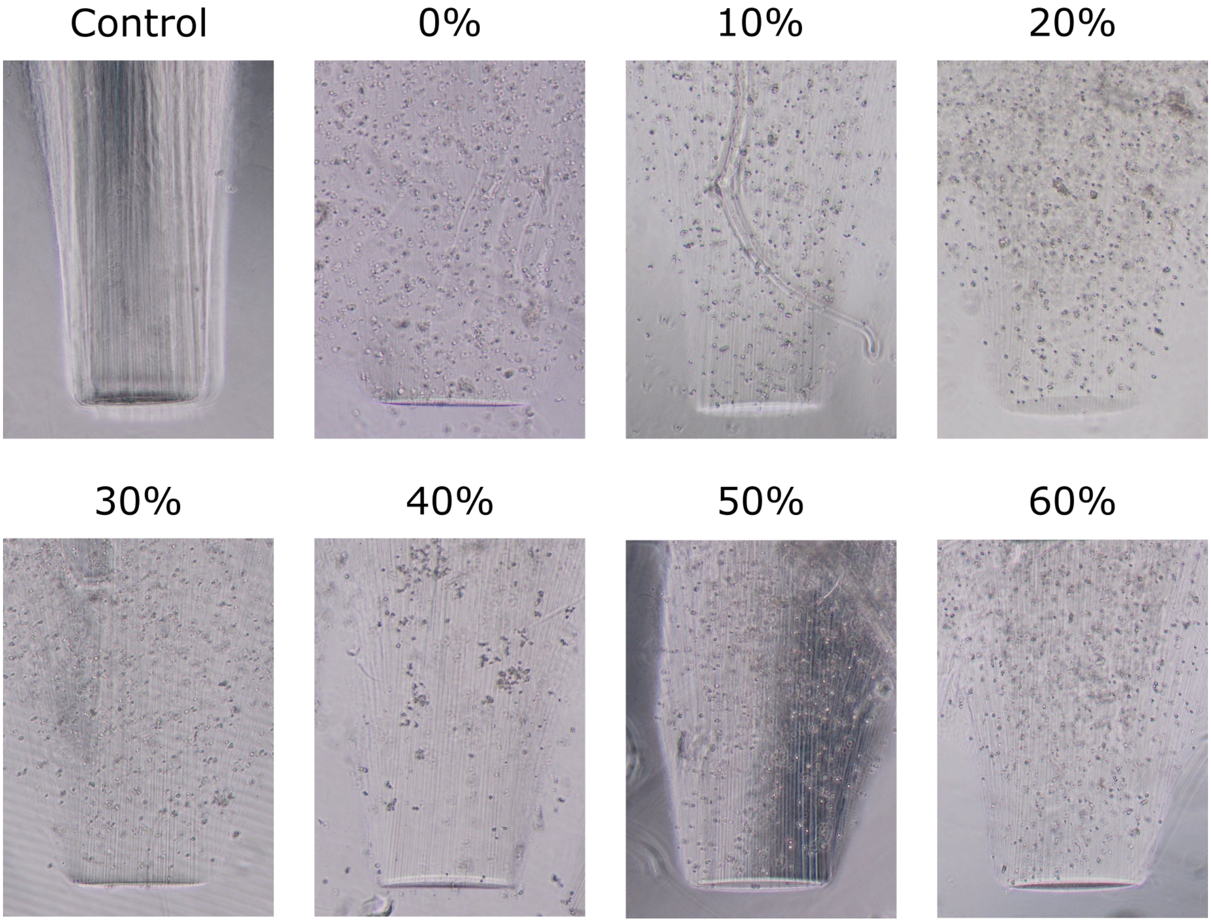

Figure S1: Formation of microstructured hydrogels at different iodixanol concentrations, and the influence of cell-induced light scattering on light propagation. Circular images (ø 500 µm) were projected into a GelMA bioink (5 x 10^6 cells mL^{-1}) with different concentrations of iodixanol. Adding iodixanol to GelMA bioinks reduces cell-mediated scattering. The resulting constructs were imaged using light microscopy.

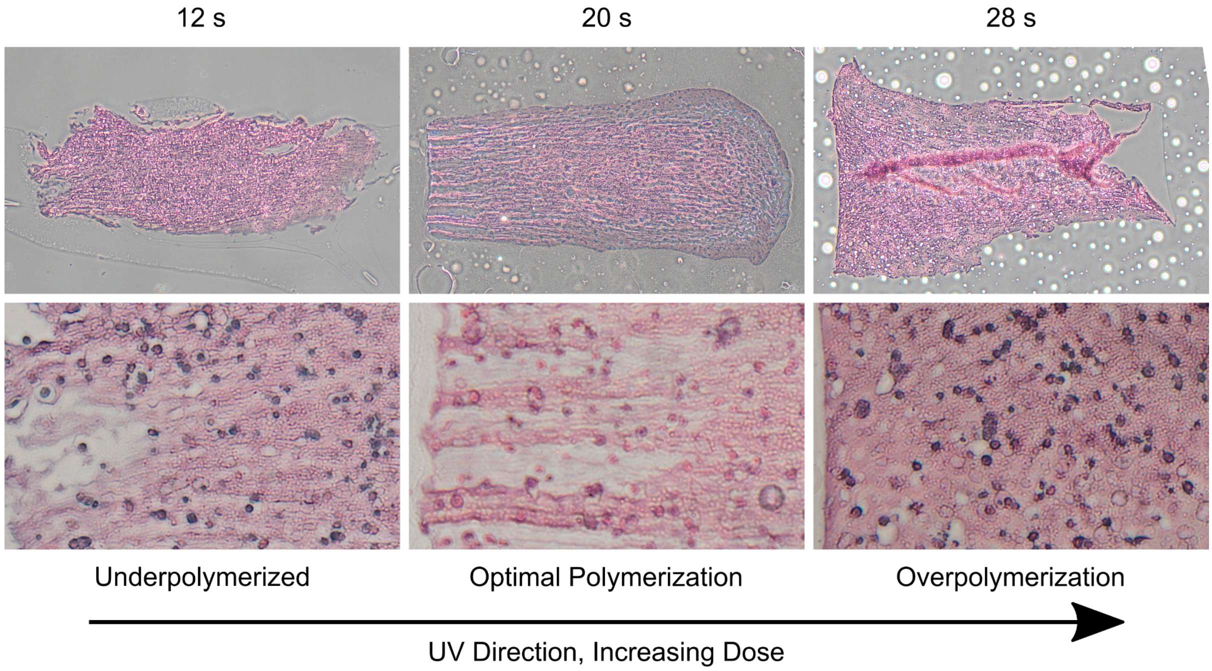

Figure S2: Histology images showing the influence of light dose and cell-induced light scattering on microstructure formation. A lower dose (reduced time) results in structure under polymerization and incomplete tissue formation. Higher dose (increased time) results in the complete polymerization of microchannels and off-target polymerization. Off-target polymerization is exacerbated by cell-induced light scattering. All tissues were printed with a high-density bioink (45 x 10^6 cells/mL cells mL^{-1}, GelMA/Iodixanol) at ≈500 mJ cm^{-2}, fixed directly after polymerization, and stained with H&E.

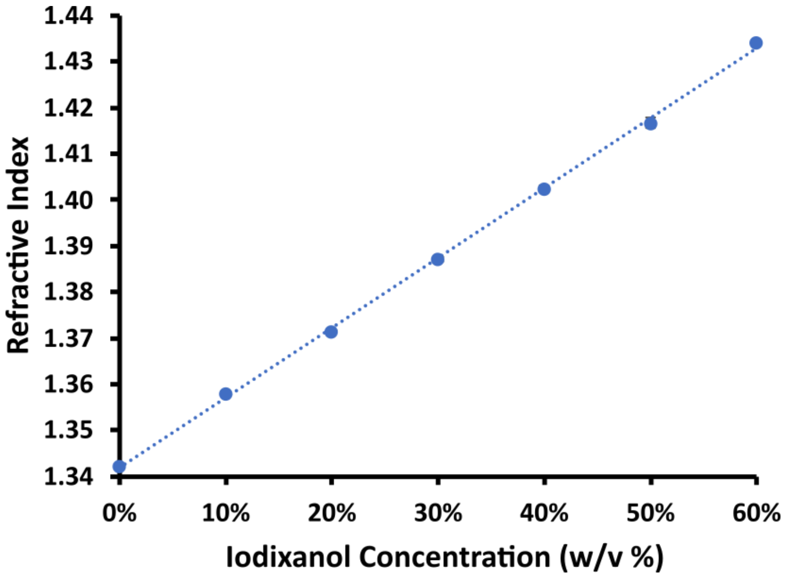

Figure S3: Adding Iodixanol to GelMA (5%) leads to a linear increase in refractive index.

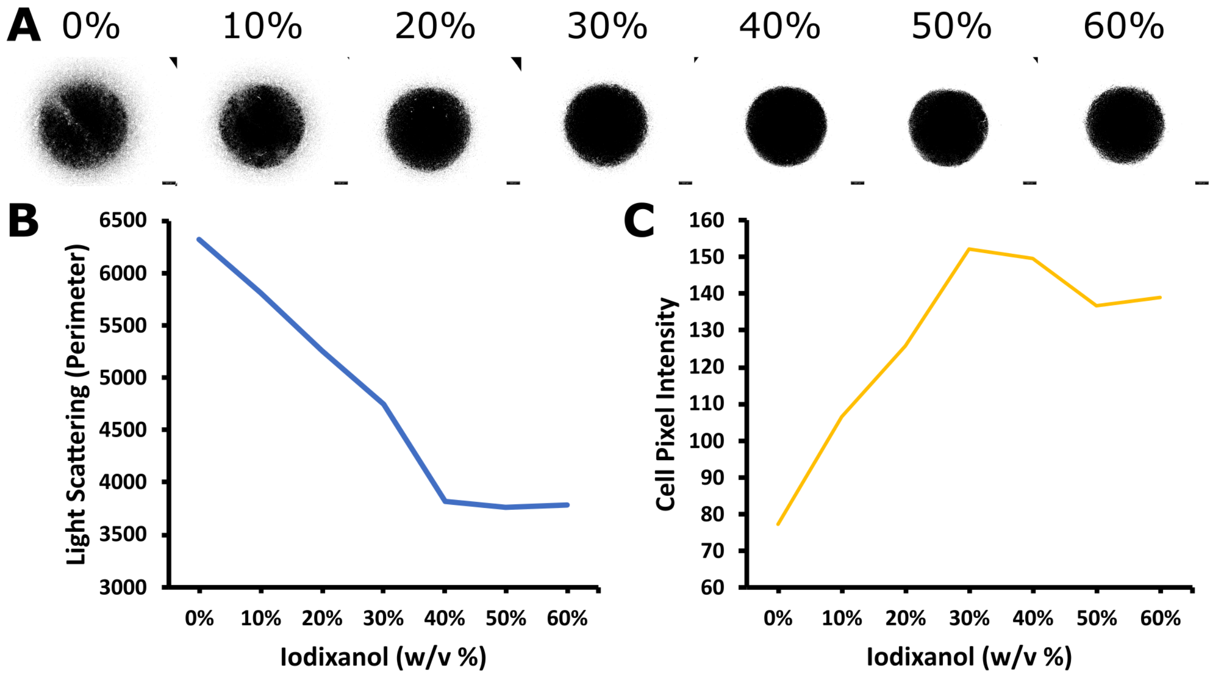

Figure S4: Refractive index matching of our bioink using iodixanol. **(A)** Light scattering of a circular image, which was projected through GelMA bioinks (5 x 10^6 cells mL^{-1}) in a 96-well plate at different concentrations of iodixanol. **(B)** Light scattering perimeter was measured at different iodixanol concentrations. **(C)** Cell pixel intensity was measured at different iodixanol concentrations.

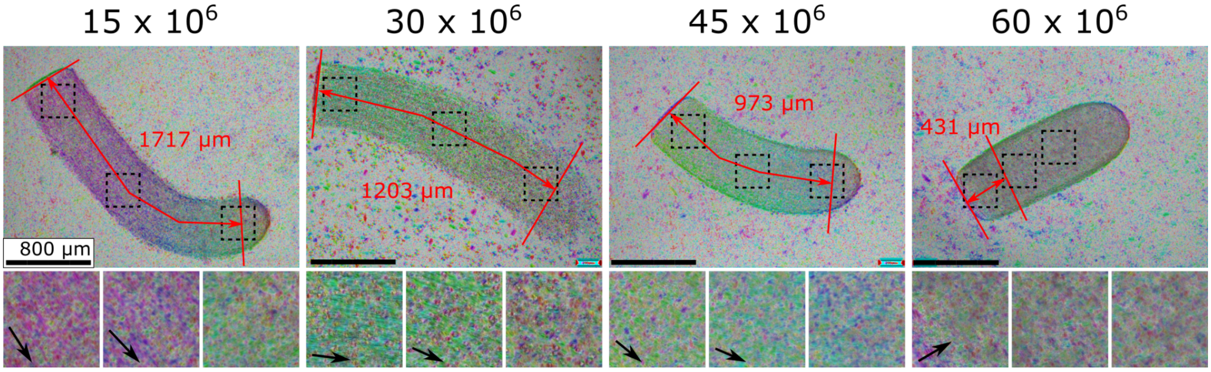

Figure S5: Alignment analysis of tissues printed at different bioink concentrations. Directional local features were highlighted on optical microscopy images using OrientationJ. Red arrows show the manually measured alignment distance. Regions from the start, middle, and end of the constructs are magnified. Black arrows show the main alignment direction (manually annotated).

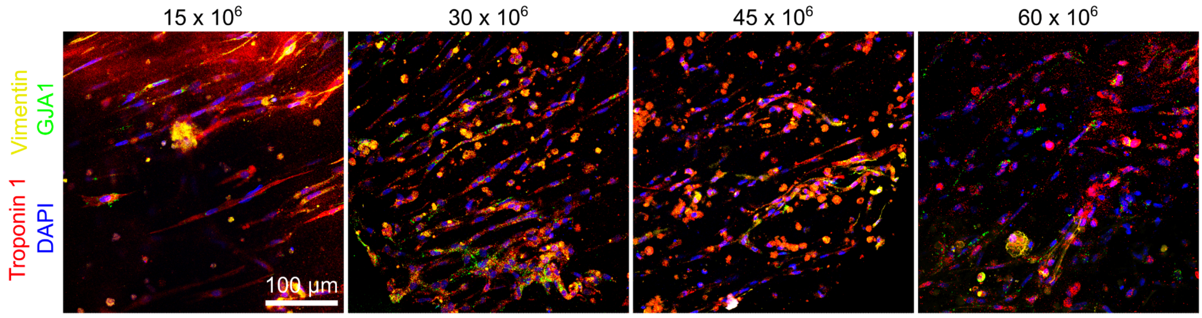

Figure S6: The effect of cellular density on cellular alignment in engineered tissues. Tissues were bioprinted at different bioink cell densities and stained for immunofluorescence (day 5). Cell-induced light scattering reduces the number of aligned cells. The quality of the images declines at higher cell concentrations due to cell-induced laser (confocal) scattering.

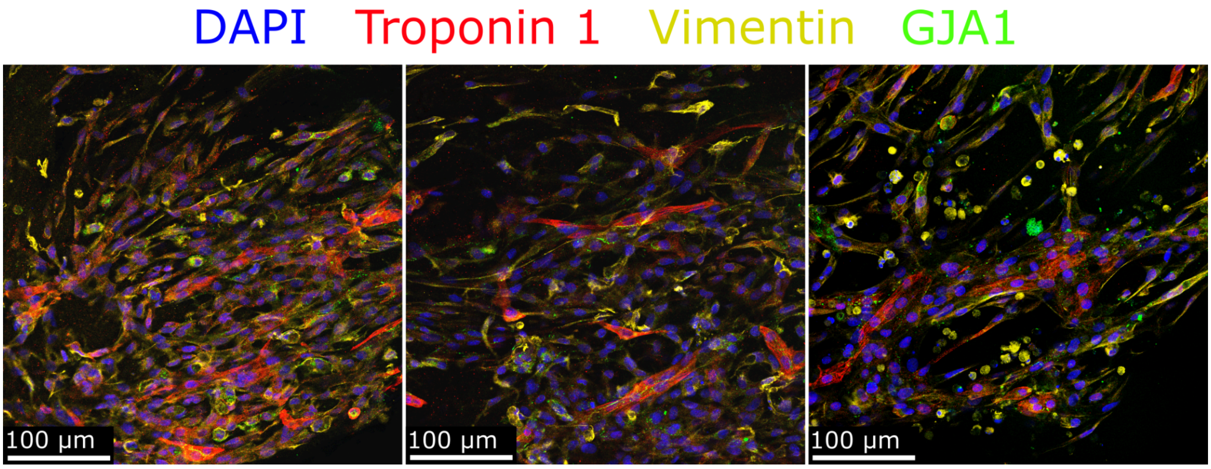

Figure S7: Immunofluorescence of different microstructured cardiac tissues showing cardiomyocyte and cardiac fibroblast morphology, bioprinted at 45 x 10^6 cells mL^{-1}.

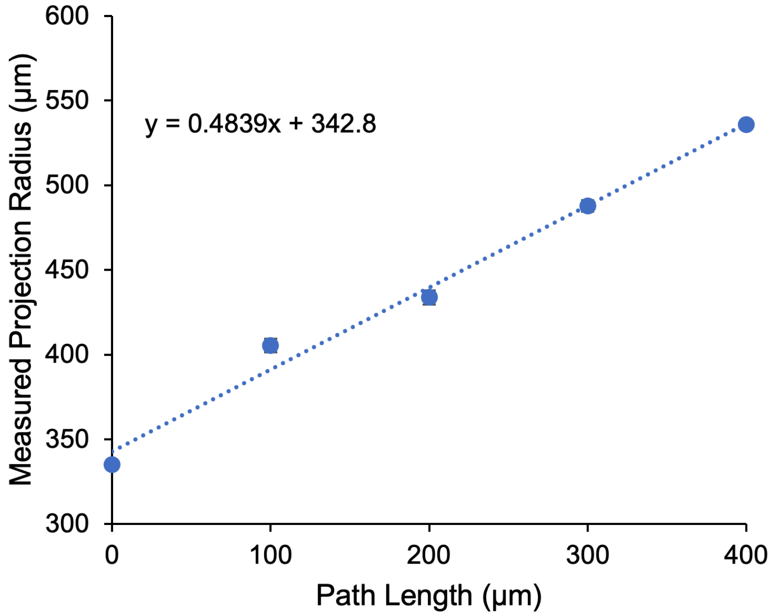

Figure S8: Decreasing image resolution with increasing light propagation distance (path length) through scattering media (cells). A circular image (ø 500 μm) was projected through the scattering media (45 x 10^6 cells mL^{-1}, GelMA (5%), and iodixanol (30%) and measured at different path lengths.

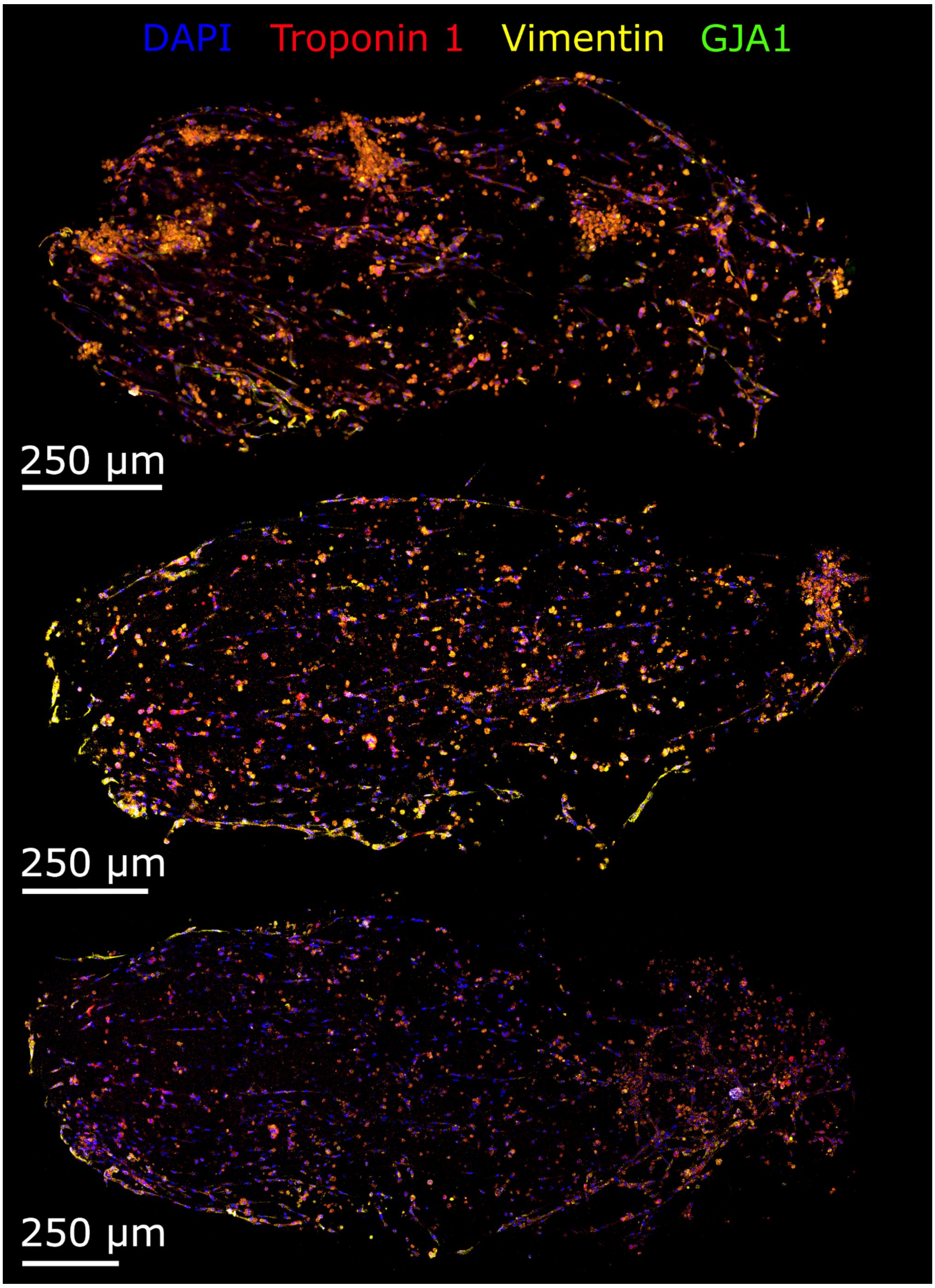

Figure S9: Immunofluorescence mosaics showing whole engineered cardiac tissues, bioprinted at 45 x 10^6 cells mL^{-1}. Cell-induced light scattering affects microstructure formation, and cell alignment, at deeper tissue depths (left to right).

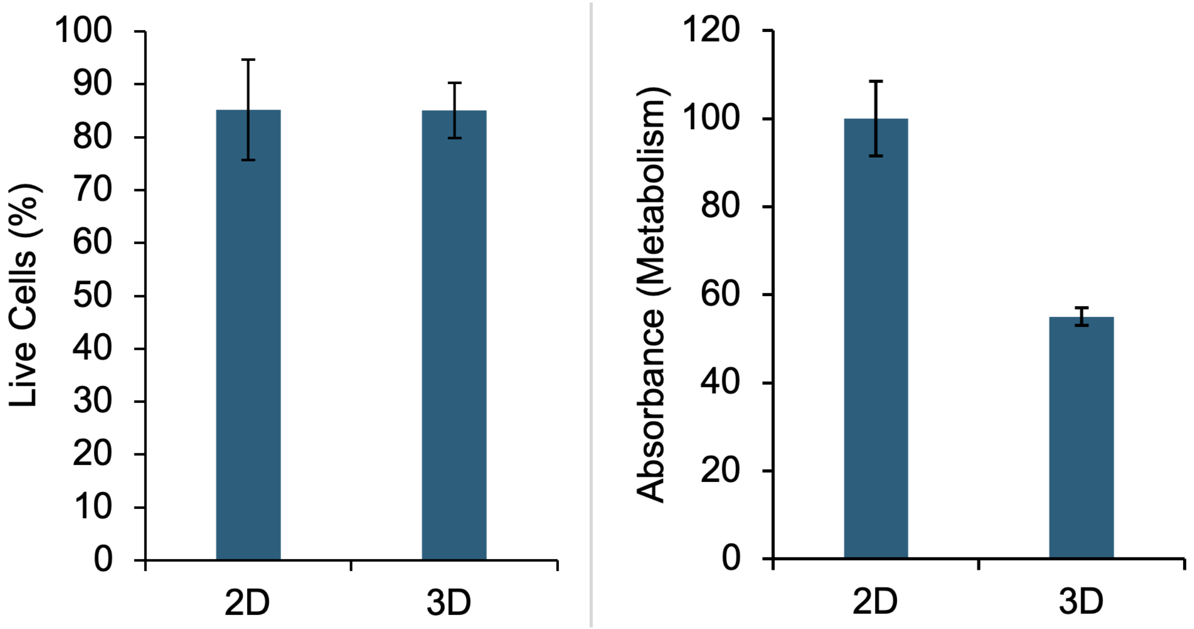

Figure S10: Cell Viability and Metabolism Assay of iPSC-CMs in biofabricated GelMA/Matrigel Hydrogels. iPSC-CMs were seeded in 2D or 3D to evaluate cell viability (Flow Cytometry) and cell metabolism (MTT Assay).

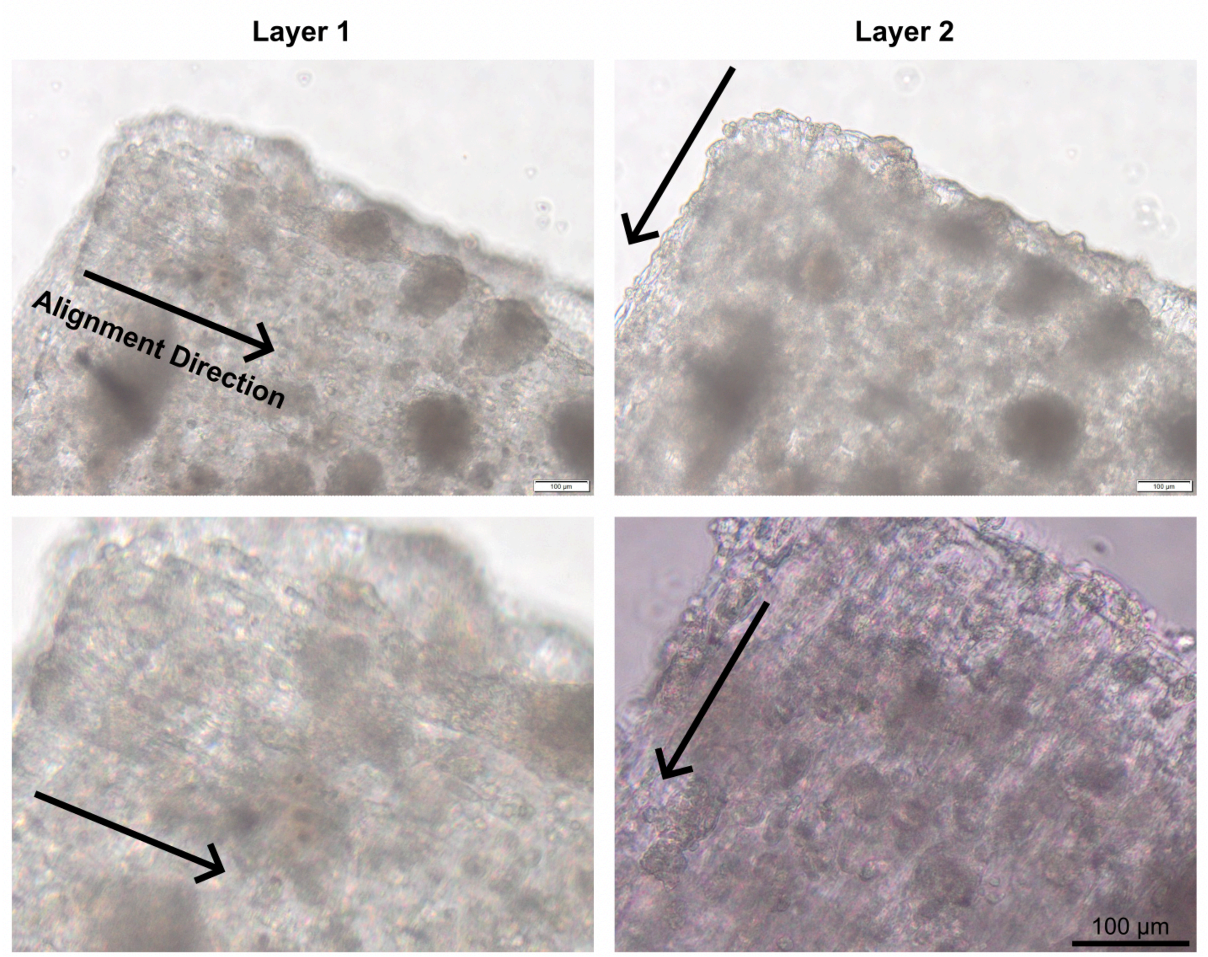

Figure S11: Optical Microscopy images show the cellular alignment direction of two-layered iPSC-CM tissue after 12 days of cell culture. Tissues were biofabricated with multidirectional light (2-layers, 90°). The corresponding cell alignment can be observed by focusing the image at different depths.

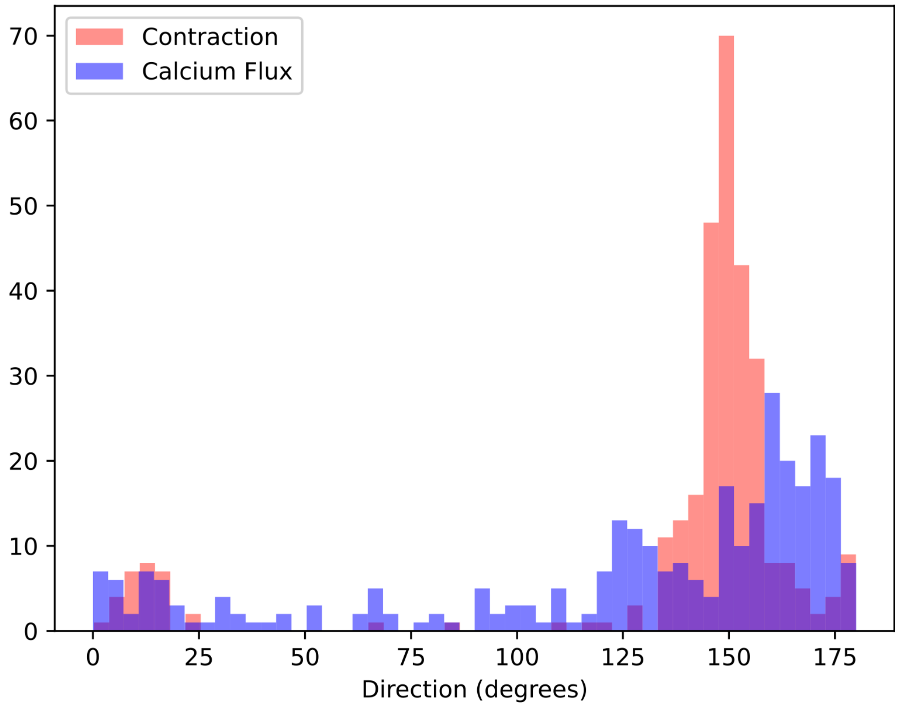

Figure S12: Comparison between the direction of impulse conduction (calcium flux) and contraction (optical flow on brightfield images). This data was generated on a multidirectional tissue (Figure 5B, and Video S4 & S6).

Table S1: Comparison of biofabrication techniques that allow for cellular alignment. The comparison includes multidirectional FLight biofabrication.

Cellular Alignment Mechanism	Cell Density	Post-Seeded or Fabricated with Cells?	Cell Alignment Possible?	Advantages	Disadvantages	References	
Micropatterning	Near native (10^7), no exact limit	Thin films only: Post-seeded Scaffolds	Yes: 2D only	• Simple, Robust and Repeatable Technique	• Requires Prior Substrate Surface Patterning<br>• Limited to Surface Level Alignment<br>• Not Scalable	A tissue-engineered jellyfish with biomimetic propulsion	1
Focused Rotary Jet Spinning	Near native (10^7-10^8), no exact limit	Thin film-like tissues: Post-seeded Scaffolds	Yes: Multidirectional 3D Alignments	• Guides cell alignment in true 3D structure<br>• Mechanical reinforcement can aid function and integration of ECTs<br>• Can create 3D large-scale ECTs	• Post-Seeding of Cells, Tissue Depth Unclear, Limited cell density (for 3D)<br>• Complex fabrication process	Recreating the heart's helical structure-function relationship with focused rotary jet spinning	2
Shear Induced Alignment (structural elements)	Near native (10^7-10^8), no exact limit	Both methods work	Yes: Multidirectional Planar Alignments	• Multidirectional alignment patterns possible<br>• Can enhance performance of cardiac tissue (self-organization)	•Only Planar Alignments, but 3D theoretically possible<br>• Scalability limited (extrusion techniques)<br>• Requires additives (structural elements)<br>• Simultaneous alignment of structural elements and cell printing is difficult	Fiber-infused gel scaffolds guide cardiomyocyte alignment in 3D-printed ventricles, Carbon nanotube-incorporated collagen hydrogels improve cell alignment and the performance of cardiac constructs	3.4
Shear Induced Alignment (organoids)	Near native (10^7-10^8), no exact limit	Both methods work	Yes: Multidirectional Planar Alignments	• High Density 3D Printed Tissue<br>• Highly Scalable (cell number)<br>• Compatible with Mechanical Stimulation Apparatus (micropillars)	• Only Planar Alignments, but 3D theoretically possible<br>• Multi-step technique: requires organoid preparation<br>• Technically demanding approach	Programming Cellular Alignment in Engineered Cardiac Tissue via Bioprinting Anisotropic Organ Building Blocks	5
Mechanical Stimulation (Strain-Induced Alignment)	Near native (10^7-10^8), no exact limit	Both methods work	Yes: 2D & 3D, but limited geometries	• Enhances electromechanical function and cell maturity<br>• Somewhat scalable approach	•Hard to generate multidirectional alignment<br>• Complexity in maintaining uniform alignment in large constructs, and over time	Tissue-engineered cardiac patch for advanced functional maturation of human ESC-derived cardiomyocytes	6
Electrical Stimulation	Near native (10^7-10^8), no exact limit	Both methods work	Yes: 2D & 3D, but unidirectional	• Benefits for electromechanical properties and cellular maturity<br>• Modular and Scalable Design	• No complex alignments possible (field-based)<br>• Fabrication process is complex<br>• Field-based approach so limited to unidirectional alignments	Biowire: a platform for maturation of human pluripotent stem cell–derived cardiomyocytes	7
FRESH-Approaches	Near native (10^7-10^8), no exact limit	Both methods work	Not shown	• Supports fabrication of complex and large 3D tissues (support bath)<br>• Supports a large range of hydrogels	• No current method for cellular alignment<br>• Limited Scalability (parallelization)	FRESH 3D bioprinting a contractile heart tube using human stem cell-derived cardiomyocytes, Direct 3D-Bioprinting of hiPSC-Derived Cardiomyocytes to Generate Functional Cardiac Tissues	8
Acoustic	Limited (10^6-10^7)	Both methods work	Yes: 2D & 3D but limited geometries	• Suitable for aligning cells without adding exogenous additives<br>• Rapid Cell Positioning and Alignment	• Viscosity constraints can limit hydrogel compatibility<br>• Limited number of defined geometric patterns<br>• Forms tissues without homogenously distributed cells	Bioacoustic-enabled patterning of human iPSC-derived cardiomyocytes into 3D cardiac tissue, Engineering Anisotropic Muscle Tissue using Acoustic Cell Patterning	9, 10
Magnetic	Near native (10^7-10^8), no exact limit	Both methods work	Yes: 2D and 3D, but unidirectional	• Can be achieved without specialist equipment<br>• Works with a variety of hydrogels	• No complex alignments possible (field-based)<br>• Requires the addition of magnetic particles<br>• Preparation of aligning agents can be time-consuming<br>• Field-based technique, so only unidirectional alignments possible	3D Magnetic Alignment of Cardiac Cells in Hydrogels, Synthetic 3D PEG-Anisogel Tailored with Fibronectin Fragments Induce Aligned Nerve Extension	11
FLight	Limited (10^6-10^7)	Both methods work	Yes: Multidirectional Planar Alignments	• Fast, and highly scalable (size, and parallel)<br>• Complex 3D geometries possible<br>• Requires no specialized equipment (laser only), or additives	• Currently only Planar Alignments (but 3D theoretically possible)<br>• Cell scattering limits resolution<br>• Compatible with a range of (photo)hydrogels, but viscosity constraints	Filamented Light (FLight) Biofabrication of Highly Aligned Tissue-Engineered Constructs	12

Text S1: GelMA/Iodixanol Refractive Index Measurement

The refractive index was measured by adding GelMA (5%) to Iodixanol (Optiprep, STEMCELL Technologies) at various concentrations in PBS. The refractive index was measured using a digital refractometer (KERN ORM 1RS, KERN & SOHN GmbH).

Text S2: Iodixanol Concentration Optimization

GelMA:Iodixanol concentration was optimized by measuring visible light scattering via light microscopy (Olympus CKX41, Olympus Schweiz AG). Primary cardiac cells (cardiomyocyte enriched) were added to GelMA bioinks (5 x 10^6 cells mL^{-1}) with different concentrations of iodixanol. We used a 4x objective lens (UPlanFl) to measure the scattering of a circular image projected from the condenser. The resulting images were processed with Fiji (ImageJ) using the auto-threshold function, and the perimeter of the ring was measured. In addition, cell pixel intensity was measured in Fiji.

Text S3: Biofabrication Dose Calibration

Before every experiment, a short dose calibration was necessary to adjust for minor variances in Lithium phenyl-2,4,6-trimethylbenzoylphosphinate (LAP) concentration. This dose calibration was performed by adding a minimum amount of bioink (15 x 10^6) to a UV transparent cuvette (UVette, Eppendorf) and projecting the desired shape (cylinder, ⌀ 500 µm). Unpolymerized GelMA was removed by washing it with warm PBS. The construct was then inspected: low doses of GelMA resulted either in no construct or an under-polymerized construct (> 1.5 mm). High doses of GelMA resulted in an over-polymerized construct. Adjustments at different cell bioink concentrations were made to isolate polymerized scaffolds of the desired length (2 mm). To minimize variance in LAP concentration, LAP was added from a stock solution (30 mg mL^{-1}). The minimum dose to achieve the desired scaffold (2 mm) length was always used.

Text S4: Alignment Analysis

Images were first acquired using light microscopy (2.5x objective). Images were imported into Fiji and rotated into a horizontal orientation. Next, OrientationJ (EPFL) (OrientationJ Analysis, default settings) was used to highlight directional local features. These images were used to identify the transition from aligned (ordered) microstructures to unaligned (disordered). The distance between the start of the scaffold and the transition point was then measured.

Text S5: Optical Scattering Simulation

Optical simulations were performed using FRED Optical Engineering Software (Photon Engineering, USA) using a Mie scattering model with the following input parameters: beam radius (340 µm), wavelength (405 nm), particle size (4.04-7.56 µm, gaussian distribution), mean free path (0.036778 mm), scattering particle index (1.38), immersion media index (1.39) and beam divergence (22.5°). The mean free path was calculated from the volume fraction of the cells. The beam divergence, immersion media index, and particle size were measured from experimental data. Simulations were developed for the different printing modalities by considering the orientation of UV light (unidirectional: light from one direction; bidirectional: light from both directions; non-directional: light from a cylindrical source).

Text S6: AFM Measurements

AFM nanoindentation was performed using a Flex-Bio AFM (Nanosurf, Switzerland) on tissues at

Day 0. The tissues were placed on positively charged glass slides (Superfrost, Thermo Fisher Scientific) to achieve adequate adhesion during measurement. PBS was used to immerse the sample. The tissue was indented using a colloidal probe consisting of a soft cantilever with a nominal spring constant (k = 0.1 N m^{-1}) and a borosilicate glass bead (⌀ 10 μm) attached to a cantilever tip (CP-qp-CONT-BSG-B-5; Nanosensors). The spring constant was obtained using the Sader method. To obtain the deflection sensitivity, the deflection displacement was recorded during the indentation of a glass slide. The AFM was mounted on top of an inverted microscope (Nikon Eclipse Ti-E) to allow for sample visualization and macroscopic positioning of the probe on the desired region of interest.

Measurements were performed starting at approximately 100 μm from the beginning of the tissues and at 200 μm intervals thereafter. The deflection and displacement of the cantilever were measured, and a force-displacement curve was produced for each location. Typically, eight 50 μm^2 regions (5 x 5 measurement grid) were indented at 200 μm intervals within the tissue. The apparent modulus (*E*) was obtained from force-displacement curves by fitting them to the Hertz contact model for a sphere indenting a semi-infinite half-space:

$$F = \frac{4}{3} \cdot \frac{E^*}{1 - v^2} \cdot R^{\frac{1}{2}} \cdot \delta^{\frac{3}{2}}$$

In the Hertz model, *F* is the force applied by the cantilever, *E** is the elastic modulus, *v* is the Poisson's ratio, *R* is the radius of the spherical bead, and δ is the indentation. For consistency with the literature and simplification purposes, the Poisson's ratio used was 0.5, equivalent to that of an isotropic, incompressible material. Therefore, *E* should be interpreted as an apparent modulus. Each curve was fitted using a custom-built Python-based algorithm to extract the *E*. Force-displacement curves without a clear contact point were discarded.

Text S7: Force Calculation

By using the measured apparent modulus (1.25 KPa) and average linear tissue contractility (27 μm), we can calculate the force output using Hooke's Law: F = kΔL, which can be rearranged as

$$F = YA\frac{\Delta L}{L_0},$$

Tissue cross-sectional area = $\pi(0.3\ mm)^2 = 0.2826\ mm^2$

Force = Young Modulus x (CSA Area (Ratio of change in length))

F = 1.25 KPa x (0.2826 mm^2 (0.03 mm/2 mm))

F = 1.25 KPa x (0.2826 mm^2 (0.015))

F = 1250 Pa x (0.004239 mm^2)

F = 5.29 Pa mm^2

F = 5.3 μN

Specific contractile force = 5.29/0.2826 = 0.0187 mN mm^{-2}

Text S8: Calcium Imaging

Calcium imaging was performed on iPSC-CM tissues following 14 days of culture. We used a Rhod-3 Calcium Imaging Kit (ThermoFischer) according to the manufacturer's instructions. Imaging was performed on a Nikon Ti2-E Widefield microscope and irradiated with light (λ = 555) (Spectra III light engine, Lumencor). Videos were acquired at the maximum framerate (approx. 17 FPS) using a 4x, or 10x objective. We tracked calcium flux by computing the center of the brightness density in each image frame of the contractile motion. This center is computed by a weighted average of the image pixel locations, weighted by pixel brightness. The direction of this center movement during contraction was plotted against the contractile direction (optical flow).
